# Mining of EHR for interface terminology concepts for annotating EHRs of COVID patients

**DOI:** 10.1186/s12911-023-02136-0

**Published:** 2023-02-24

**Authors:** Vipina K. Keloth, Shuxin Zhou, Luke Lindemann, Ling Zheng, Gai Elhanan, Andrew J. Einstein, James Geller, Yehoshua Perl

**Affiliations:** 1 School of Biomedical Informatics, University of Texas Health Science Center at Houston, Houston, TX USA; 2 Department of Computer Science, New Jersey Institute of Technology, Newark, NJ USA; 3 School of Medicine and Health Sciences, The George Washington University, Washington (D.C.), USA; 4 Computer Science and Software Engineering Department, Monmouth University, West Long Branch, NJ USA; 5 Renown Institute for Health Innovation, Desert Research Institute, Reno, NV USA; 6 Cardiology Division, Department of Medicine, Columbia University Irving Medical Center, New York, NY USA; 7 Department of Radiology, Columbia University Irving Medical Center, New York, NY USA

**Keywords:** Interface terminology, COVID-19 ontologies, Concept mining, EHR annotation

## Abstract

**Background:**

Two years into the COVID-19 pandemic and with more than five million deaths worldwide, the healthcare establishment continues to struggle with every new wave of the pandemic resulting from a new coronavirus variant. Research has demonstrated that there are variations in the symptoms, and even in the order of symptom presentations, in COVID-19 patients infected by different SARS-CoV-2 variants (e.g., Alpha and Omicron). Textual data in the form of admission notes and physician notes in the Electronic Health Records (EHRs) is rich in information regarding the symptoms and their orders of presentation. Unstructured EHR data is often underutilized in research due to the lack of annotations that enable automatic extraction of useful information from the available extensive volumes of textual data.

**Methods:**

We present the design of a COVID Interface Terminology (CIT), not just a generic COVID-19 terminology, but one serving a specific purpose of enabling automatic annotation of EHRs of COVID-19 patients. CIT was constructed by integrating existing COVID-related ontologies and mining additional fine granularity concepts from clinical notes. The iterative mining approach utilized the techniques of 'anchoring' and 'concatenation' to identify potential fine granularity concepts to be added to the CIT. We also tested the generalizability of our approach on a hold-out dataset and compared the annotation coverage to the coverage obtained for the dataset used to build the CIT.

**Results:**

Our experiments demonstrate that this approach results in higher annotation coverage compared to existing ontologies such as SNOMED CT and Coronavirus Infectious Disease Ontology (CIDO). The final version of CIT achieved about 20% more coverage than SNOMED CT and 50% more coverage than CIDO. In the future, the concepts mined and added into CIT could be used as training data for machine learning models for mining even more concepts into CIT and further increasing the annotation coverage.

**Conclusion:**

In this paper, we demonstrated the construction of a COVID interface terminology that can be utilized for automatically annotating EHRs of COVID-19 patients. The techniques presented can identify frequently documented fine granularity concepts that are missing in other ontologies thereby increasing the annotation coverage.

## Background

Electronic Health Records (EHRs) provide a systematic account of patient health information to authorized users in a real time setting in a secure manner. EHRs include information related to patient demographics, past medical history, medications, vital signs, tests, day-to-day progress, discharge summaries, etc. An EHR stores information in both structured forms (e.g., medication lists, numeric test results, demographic data) and unstructured text (e.g., admission notes, progress notes, discharge summaries). Extensive research has been conducted utilizing the structured part of EHRs, which is easily accessible with diagnostic/procedure/medication terms which correspond to concepts from standard terminologies. Analyzing unstructured textual data in EHRs for extracting concepts related to medical problems, procedures, treatments, etc., is a widely researched topic in clinical natural language processing (NLP) [1–10]. Conventionally, medical text is manually annotated, e.g., to generate data for training different machine learning models. Due to privacy laws, among other issues, curating a large, publicly available, expertly annotated medical text corpus is difficult. Annotating clinical text requires substantial medical expertise and is expensive and time-consuming. Using machine learning is an alternative to using human expertise, but it requires high quality training data. Situations such as the COVID-19 pandemic, where fast research is essential, accentuate the need for alternative approaches that are fast and effective for preparing training data for annotating EHRs to cope with rapidly changing scenarios.

In the absence of manually annotated datasets, off-the-shelf annotation systems [11–13] can be used by researchers and medical practitioners to annotate medical data. These annotation systems rely on biomedical terminologies and ontologies to identify, extract, and normalize concepts from clinical text. In addition, rule-based engines and curated abbreviation/acronym dictionaries are incorporated to improve the overall performance. Even though these annotation systems have demonstrated reasonable performance across various NLP tasks, they have several limitations regarding annotation of EHR notes. The quality of the annotations is highly dependent on the terminology or ontology to which the concepts are mapped. For example, concept extraction and mapping tools such as MetaMap [12] and cTAKES [13] use the Unified Medical Language System (UMLS) Metathesaurus [14] which combines terms from more than 200 source vocabularies (but is not itself a terminology) and hence is highly redundant and error-prone.

Standard reference terminologies do not contain many of the medical phrases that are frequently recorded in EHRs. For example, the COVID-19 pandemic has led to the rapid discovery of new phenomena such as the Multisystem Inflammatory Syndrome in Children (MIS-C), Post-acute sequelae of COVID-19 (PASC) known as "long COVID," and recently, the COVID-19 Associated Pulmonary Aspergillosis (CAPA). Existing terminologies and ontologies did not contain concepts for such new phenomena, whether they were general purpose terminologies such as SNOMED CT [15], or COVID-specific ontologies like CIDO [16].

In addition, the design principles of many terminologies (such as easy maintainability) do not favor including finer granularity concepts. The common radiology findings in COVID-19 infections such as *bilateral ground-glass opacities*, *parenchymal fibrotic bands*, and *interlobular septal thickening* as identified by various studies [17–19] are of fine granularity and are not represented in reference terminologies. However, they are of practical importance and correspond to "chunks" in cognitive psychology [20–22]. These chunks often include words that describe generic concepts (e.g., consolidation, lobular) and those do exist in reference terminologies. For annotating EHRs it is important to capture such chunks as they express units of thought of medical professionals writing the EHR notes. Hence, the annotations using only standard reference terminologies/ontologies are incomplete and insufficient, resulting in a loss of information. Note that many finer granularity terms used in EHRs for other specialties, are not concepts in clinical terminologies. Some such terms in cardiology which are not concepts in SNOMED CT are: *tricuspid valve endocarditis, inspiris valve, patch closure of aortic root abscess and atrial fistula*.

**Fig. 1:**
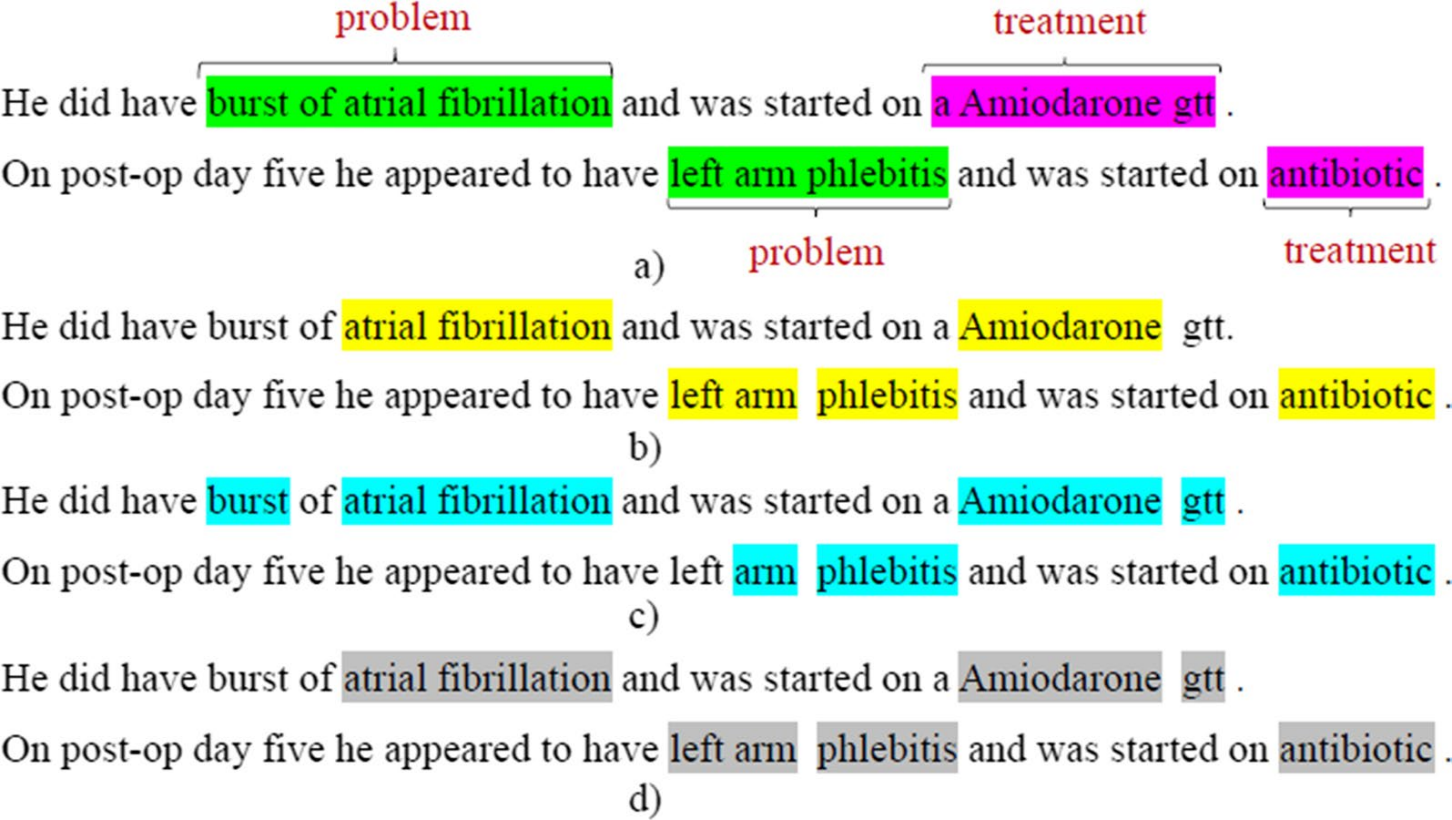
**a** Ground truth annotations from the 2010 i2b2/VA challenge, **b** annotations using NCBO Annotator with SNOMED CT as the target terminology, **c** annotations using MetaMap Lite with UMLS, and **d** annotations using cTAKES with UMLS

To further demonstrate this issue, consider Fig. 1(a), which shows the manual annotations for an excerpt from the 2010 i2b2/VA challenge [1]. Figures (b), (c), (d) show annotations using NCBO Annotator [11] with SNOMED CT, MetaMap Lite [23], and cTAKES [13] (both use the UMLS Metathesaurus). While *burst of atrial fibrillation* is the ground truth annotation, none of the above annotation systems can capture this chunk, as it is not present in the reference terminologies. This scenario highlights a gap in utilizing existing terminologies for annotating clinical corpora.

We note that “post-coordination” supported by SNOMED CT [24, 25], can be used to construct fine granularity concepts from simple ones. However, when mining a phrase, it is difficult for human experts to determine whether component concepts to be post-coordinated already exist. Automated methods for building post-coordinated expressions have had limited success [26].

In this paper, we address these issues and propose the curation of a COVID Interface Terminology (CIT) that would support the annotation of EHRs of COVID-19 patients. The design principle of interface terminology is application-oriented with a focus to maximize utilization by the end-users [35]. Thus, it can contain fine granular concepts corresponding to phrases found in EHR notes. Furthermore, interface terminologies maintain a richer synonym content and contain colloquial usages and common medical terms. Hence, CIT can be enriched by incorporating fine granularity concepts which can be mined from EHRs, since the best source for phrases for annotation of EHR notes is the EHR notes themselves.

In this paper, we describe how we constructed a COVID Interface Terminology (CIT) from radiology case studies of COVID-19 patients based on Radiopaedia, which is “a wiki-based international collaboration with the mission to create the best radiology reference” [27]. A prior preliminary study performed on a different dataset [28] explored the feasibility of our curation approach without testing its generalizability [29]. In the current paper, we demonstrate the generalizability of our approach by evaluating the CIT on a held-out test dataset of radiology case studies of COVID patients.

We used automated text mining techniques for extracting fine granularity phrases from clinical text for their addition into the CIT. Since these are brute-force techniques, a one-time review by domain experts was required before addition into the CIT. In a second phase of enriching the CIT, the concepts added to it based on text mining can serve as training data for Machine Learning (ML) models. Applying ML methods will enable further addition of more concepts into the CIT, thereby increasing the percentage of the successfully annotated text of the processed EHR notes.

The resulting CIT will enable automatic annotation of any textual COVID-related EHR note, with sufficiently high annotation coverage to improve research into the rich clinical knowledge hidden in the EHR notes. It was observed (by Wang et al. [30]) that the structured EHR data is not sufficient for identifying all the PASC symptoms, because descriptions of most symptoms are manifested in the clinical notes in a nuanced way. For developing a Post-Acute Sequelae of COVID-19 (PASC) symptom lexicon, they extracted the symptoms from clinical notes utilizing the MTERMS NLP tool [31] and the lexicon was iteratively refined and evaluated. Annotated EHR notes provide substantial support for such research where the nuanced descriptions of various aspects of a disease provide added value, compared to the use of structured EHR data alone.

We emphasize that CIT is not designed as a generic COVID-19 terminology, but as an interface terminology for one application—annotation of EHR notes of COVID-19 patients. In the current study, we set to explore with a relatively small dataset whether this approach is indeed capable of meaningfully increasing the annotation coverage. We plan a follow-up full scale study with 5000 EHR notes for which we expect a larger annotation coverage of about 75–80%, since a larger dataset will contain more commonly used terms. This sample will be composed to reflect proper age/gender/race/ethnic distribution of patients and will be selected from different time periods to reflect the developments in the variants of the COVID-19 disease and the treatments available. Such annotation will capture most of the relevant knowledge in the EHR notes and will enable extensive research into the rich knowledge hidden in the unstructured EHR notes of COVID-19 patients.

### Annotation tools

Several annotation tools have been developed to extract information from the biomedical literature and clinical text. One of the earliest attempts to use NLP methods to automatically encode data was the Medical Language Extraction and Encoding system (MedLEE) [32]. MedLEE was developed at Columbia University and extracts clinical concepts from textual data along with a set of modifiers. A group of researchers at the Brigham and Women’s Hospital and Harvard Medical School developed the Health Information Text Extraction (HITEx) [33] software, an open-source clinical NLP system that is incorporated into the i2b2 toolset. Two of the most widely used tools in the biomedical field are MetaMap [12] and cTAKES [13]. MetaMap was developed by the National Library of Medicine (NLM) and maps concepts extracted from biomedical and clinical text to concepts in the Unified Medical Language System (UMLS). The Clinical Text Analysis and Knowledge Extraction System (cTAKES) [13], uses a combination of rule-based and machine learning techniques to extract and normalize concepts for inclusion into the UMLS.

QuickUMLS [34] is yet another tool that maps extracted concepts to concepts in the UMLS Metathesaurus. NCBO Annotator [11] takes a broader approach by enabling concepts extracted from text to be mapped to any ontology that is uploaded on BioPortal [35]. Another set of annotation tools such as PubTator [36] and BERN [37] focuses on identifying only specific entity types like genes, diseases, chemicals, etc. Tools such as CLAMP [38] that are trained with manually annotated datasets using machine learning and deep learning techniques can support the annotation of a wide variety of entity types based on the training set.

### Interface terminologies

Reference Terminologies (RTs) have been widely used in healthcare to support billing, insurance, data analysis, and decision support systems. However, RTs often fail to represent information with the same granularity and clarity as documented by physicians in EHR notes. Thus, Interface Terminologies (ITs) were developed to “bridge the gap between information that is in the physician’s mind and information that can be interpreted by computer applications.” [39] They contain clinical jargon, colloquial usages and are richer in synonym content than RTs. The differences between the two have been a subject of extensive research [40–42]. Rosenbloom et al. [43–48] defined several evaluation metrics such as quantity and quality of assertional knowledge, adequacy of synonymy, balancing pre-/post-coordination, etc. for improving and evolving interface terminologies. One recommended approach [44] is to start the construction of an interface terminology from an existing reference terminology or its subhierarchies rather than starting from scratch. Following this recommendation, we constructed the initial version of CIT by integrating existing COVID ontologies/terminologies.

### Existing COVID-19 ontologies/terminologies

To expedite research on COVID-19, and to support extraction, linking, and standardization of coronavirus-related entities, a number of COVID-19 ontologies have been developed. NCBO BioPortal [44] hosts several of them. The largest among them is the Coronavirus Infectious Disease Ontology (CIDO) [16] developed with the aim to provide a standardized “representation of various coronavirus infectious diseases, including their etiology, transmission, pathogenesis, diagnosis, prevention, and treatment.” [16, 49] CIDO ensures interoperability by following the OBO Foundry [50] principles and integrating concepts from about 20 other ontologies such as ChEBI [51], UBERON [52], GO [53], NDF-RT [54], and HPO [55]. The latest release of CIDO on BioPortal contains 8775 classes (concepts) and continues to be updated. For summarization and visualization of CIDO see [56].

Another COVID-19 related ontology on BioPortal is the COVID-19 ontology [57] (2270 concepts). It covers concepts related to cell types, genes, and proteins involved in virus-host-interactions, as well as medical and epidemiological concepts relevant to COVID-19 [57, 58]. Compared to CIDO, it includes more concepts related to various symptoms caused by the disease.

Two more moderately sized COVID-related ontologies are the COVID-19 Infectious Disease Ontology (IDO-COVID-19) [59] (486 concepts) and the World Health Organization’s (WHO) COVID-19 Rapid Version CRF semantic data model (COVIDCRFRAPID) [60] (398 concepts). IDO-COVID-19 is an extension of the Infectious Disease Ontology (IDO) [61] and the Virus Infectious Disease Ontology (VIDO) [62]. Additionally, BioPortal contains two small ontologies, the COVID-19 Surveillance Ontology (COVID19) [63] (32 concepts), and the COviD-19 Ontology for Cases and Patient information (CODO) [64] (90 concepts).

Besides the ontologies available on BioPortal, another open-source ontology is the ACT COVID Ontology v3.0 (2446 concepts) that is accessible via GitHub [65] as set of SQL files. This ontology supports cohort identification and related research by incorporating terms related to diagnosis, procedures, and medication codes from ICD [66], LOINC [67], CPT [68] and NDC [69]. In addition to these, the UMLS, SNOMED CT [15], and LOINC contain lists of concepts related to COVID-19 on their respective websites that were incorporated over time.

### Dataset–radiology case studies

As alluded to above, large publicly available annotated datasets are a scarce resource in the clinical domain. While corpora of scientific papers related to COVID-19 [70, 71], annotated COVID-19 clinical trial corpus [72], and database sharing statistics of EHR prevalence rates [73] are available, we are not aware of a publicly available annotated clinical text corpus for COVID-19. A study has been performed on extracting COVID-19 diagnoses and symptoms from clinical text, but the dataset is not yet publicly available [74]. Hence, in this study, we used radiology case studies of COVID-19 patients available on Radiopaedia [27]. Radiology reports including Computed Tomography (CT) scans and X-rays play an essential role in the diagnosis and treatment of COVID-19. For this research, we used COVID-19 radiology case studies posted on the Radiopaedia.org website [27]. Each case study includes the patient’s age and sex, medical history, symptoms of the presentation, detailed chest CT and X-ray illustration and descriptions, diagnosis, and discussion [27].

## Materials and methods

### Dataset

We extracted 166 radiology case studies of COVID-19 patients from Radiopaedia [27]. Radiopaedia guidelines for uploading case studies include detailed instructions on copyright, plagiarism, and de-identification of both textual and image data following HIPAA guidelines. For our study, we collected only textual information, which includes diagnosis, study findings, and a detailed discussion section. We used a random sample of 132 (80%) case studies (*DS*_*build*_) for building the CIT and reserved the remaining 34 (20%) case studies (*DS*_*test*_) for testing. We evaluated our methods by measuring the performance that would be obtained when annotating *DS*_*test*_, the part of the sample that was not utilized for the construction of CIT.

### Building COVID interface terminology (CIT)

**Fig. 2:**
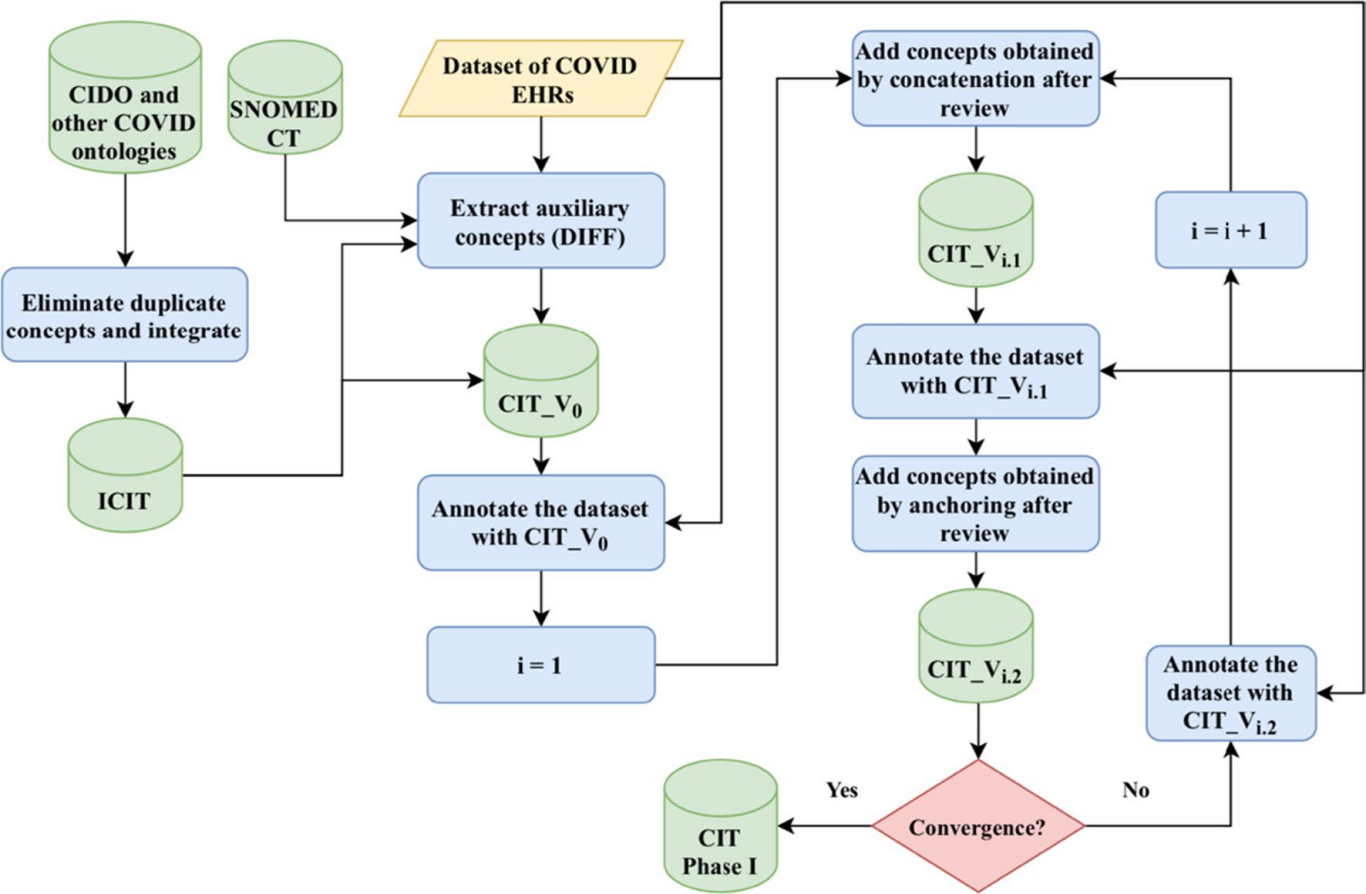
A flowchart of the steps involved in building the COVID Interface Terminology (CIT)

An overview of the proposed approach for building the CIT is illustrated in the flowchart of Fig. 2. The approach consists of three steps: (1) Creating an initial CIT, (2) Adding auxiliary concepts to the initial CIT, and (3) Mining chunks to enrich the CIT. Below we describe the steps in detail.

### Creation of initial COVID interface terminology (ICIT)

Several COVID-related ontologies have been made available on repositories such as BioPortal. Instead of starting from scratch, we integrated concepts from these ontologies to create the Initial CIT (ICIT). The Coronavirus Infectious Disease Ontology (CIDO) is the largest publicly available COVID-related ontology. It contains concepts related to the etiology, transmission, diagnosis, treatment, etc., of COVID-19. CIDO also incorporates several relationships including *part of*, *occurs in*, *treatment for*, *regulated by*, and *infects*. Hence, we used CIDO as the basis and added concepts from the following ontologies: COVID-19, IDO-COVID-19, COVIDCRFRAPID, COVID19, and CODO. We note that many of these ontologies follow the OBO Foundry principles and hence have a significant overlap among their concepts. During the integration process, we eliminated these duplicate concepts. In addition to the above-mentioned ontologies, we also added COVID-related concepts from the ACT COVID ontology, SNOMED CT, LOINC, and the UMLS. The synonyms of a concept across different ontologies were grouped under a single concept ID. For example, some of the synonyms of the concept COVID-19 are *COVID19*, *SARS-CoV-2 infection*, *2019 Novel Coronavirus (2019-nCoV) infection*, and *Disease caused by 2019-nCoV*. To automate the elimination of duplicate concepts and identification of synonyms of existing concepts, the concepts from the above-mentioned ontologies were added, one at a time, maintaining a list of all existing concepts and performing a string match (exact/fuzzy) whenever a new concept is added. Exact matching helps to eliminate duplicate concept names, while fuzzy matching helped to identify possible synonyms. These synonyms were manually reviewed and were grouped under respective concept IDs.

### Adding auxiliary concepts to ICIT

The Centers for Disease Control and Prevention (CDC) has determined that patients with certain preexisting medical conditions are more likely to get severely ill from COVID-19 [75]. Some examples of such co-morbidities are cancer, chronic kidney disease, chronic lung disease, diseases of the immune system, dementia, diabetes, cardiovascular diseases, and obesity, as well as smoking and substance abuse disorders. Similar issues exist for patients of advanced age, with accumulating research showing that different variants of the coronavirus affect different age groups differently. Information regarding these co-morbidities and conditions is often scattered over different sections in the EHR notes such as prior medical history and social history. Even though these conditions are not symptoms of COVID, it is important to identify and extract the corresponding concepts from EHR notes and add them to CIT, as they play a major role in providing precautions and preventive care and in determining the severity and recommended course of treatment for high-risk patients. We used SNOMED CT [15], the most comprehensive clinical terminology, for identifying these auxiliary concepts and for enhancing ICIT with them.

**Fig. 3:**
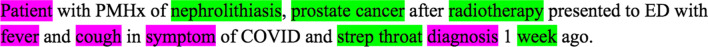
An excerpt from the EHR of a COVID patient with concepts present in ICIT (highlighted in pink) and auxiliary concepts from SNOMED CT missing in ICIT (highlighted in green)

In Fig. 3, we show an excerpt of EHR notes where the concepts present in ICIT are highlighted in pink and the auxiliary concepts present in SNOMED CT (but not present in ICIT) are highlighted in green. The auxiliary concepts include the patient’s prior medical conditions such as *nephrolithiasis* and *prostate cancer* and other clinical concepts such as *strep throat*, and *radiotherapy*. Yet another type of auxiliary concepts appearing in clinical notes include generic terms related to temporal information (e.g., time, duration, frequency) such as *week*, *monthly*, *per hour*, etc.

The process of identifying auxiliary concepts for enhancing ICIT involves the following steps: (1) Annotate *DS*_*build*_ with concepts from ICIT, (2) Annotate *DS*_*build*_ with concepts from SNOMED CT, (3) Identify a list of concepts that are annotated by SNOMED CT but not by ICIT to extract auxiliary concepts. For this, we created a program to calculate the DIFF (set difference) between the text segments annotated with SNOMED CT and those annotated with ICIT:

1 $$DIFF \, = \, \left\{ {DS_{build} \;annotated\;with\;SNOMED\;CT} \right\} \, {-} \, \left\{ {DS_{build} \,annotated\;with\;ICIT} \right\}$$

(4) Organize the DIFF concepts into separate hierarchies, according to their kinds, and integrate them with ICIT to form the zeroth version of CIT (CIT_V_0_). The details regarding the annotation process are described below.

*Annotation:* To support our methodology, the annotation tool used needs to satisfy the following requirements. Firstly, we need a general-purpose tool that supports all entity types and is not restricted to just disease, chemicals, genes, etc. Secondly, manually annotated public EHR datasets are not available for COVID-19 and hence annotation tools that require annotated datasets for training cannot be used. Finally, we use an iterative approach to build the CIT, creating several versions along the way while annotating *DS*_*build*_ with each version to extract new phrases that could correspond to medical concepts. Hence, we require a tool that can annotate text using concepts from different versions of CIT that we created. Several systems satisfy the first two requirements (e.g., MetaMap, QuickUMLS, cTAKES), but fail to meet the final requirement as these systems are designed to use concepts from the UMLS Metathesaurus for annotation.

We use the NCBO Annotator [11] as our annotation tool, because it fulfills all three requirements. It is possible to upload new terminologies into NCBO BioPortal [35]. These new terminologies can then, in turn, be used by the NCBO Annotator for annotating text. Thus, we uploaded consecutively better versions of the CIT into BioPortal and used each version in turn to annotate *DS*_*build*_ to derive the next version of CIT.

### Mining chunks to enrich CIT

To extract fine granularity phrases (chunks) that are not present in CIT_V_0_, we used two operations—concatenation and anchoring.

**Fig. 4:**
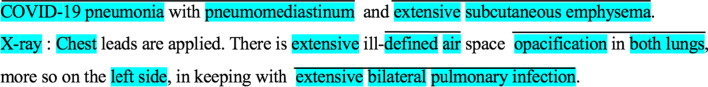
An excerpt from a Radiopaedia case study [27] showing phrases obtained by concatenation, represented by overbars, of existing concepts in CIT_V_0_ (highlighted)

*Extracting Phrases by Concatenation:* To apply concatenation, we first annotate *DS*_*build*_ with CIT_V_0_. We observed that fine granularity phrases often contain one or more generic concepts that are already present in reference terminologies. Concatenation combines two or more concepts that are adjacent to each other in *DS*_*build*_ to form a fine granularity phrase that we propose as a fine granularity concept. If there are stop words between two adjacent concepts in *DS*_*build*_*,* we also include them in the concatenated phrase. In Fig. 4, we show, using overbars, all possible phrases that are obtained by concatenation of annotated CIT_V_0_ concepts (highlighted). For example, the phrase *extensive bilateral pulmonary infection* is obtained by concatenating three generic concepts—*extensive*, *bilateral*, and *pulmonary infection*. *Opacification in both lungs* is an example where stop words are included.

All the phrases obtained by concatenation along with their context (the sentence in which each phrase appears) are manually reviewed to filter out the phrases that should be included in the CIT. We note that since concatenation is a brute-force technique, not all the phrases obtained are candidates for inclusion into the CIT (e.g., *defined air* in Fig. 4). During the review process, such phrases are eliminated and added to a list of rejected phrases so that they are not considered for review in the subsequent iterations. This reduces the number of phrases to be reviewed in the following iterations considerably. The review process is discussed in detail under subheading “Review Process” below. The accepted phrases are added to CIT_V_0_ to obtain CIT_V_1.1_.

*Extracting Phrases by Anchoring:* Anchoring is applied after annotating *DS*_*build*_ using NCBO Annotator with concepts from CIT_V_1.1_ (which includes the accepted phrases obtained by concatenation). Anchoring extracts phrases by adding an unannotated word to the left, right, or on both sides of an already existing concept in the annotated *DS*_*build*_. As with concatenation, we allow stop words in between. We represent the anchoring operation using the three rules below:

$${\text{Rule 1}}:{\text{ w1 }} + {\text{ sw}}^{*} \, + \, \left[ {\text{annotated concept}} \right]$$

$${\text{Rule 2}}: \, [{\text{annotated}}\,{\text{concept}}] \, + {\text{ sw}}^{*} \, + {\text{ w1}}$$

$${\text{Rule 3}}:{\text{ w1 }} + {\text{ sw}}^{*} \, + \, [{\text{annotated}}\;{\text{concept}}] \, + {\text{ sw}}^{*} \, + {\text{ w2}}$$

In these rules, we define sw* (* denotes the Kleene star [76]) to mean 0 or more stop words and w1 and w2 are unannotated words appearing to the left or right of an annotated concept.

**Fig. 5:**
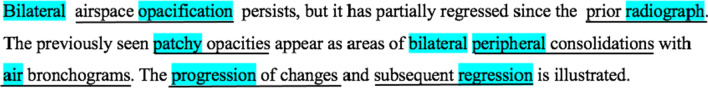
An excerpt from a Radiopaedia case study [27] indicating phrases obtained by anchoring by underlines. Annotated concepts are highlighted

The phrase *airspace opacification* (Fig. 5) is an example of a phrase obtained by applying Rule 1, where a left word is added to the existing concept *opacification*. Similarly, *air bronchograms* and *progression of changes* are example phrases obtained by applying Rule 2, with the stop word "of" included in the last example.

As for concatenation, all the phrases obtained by anchoring, along with their context, are passed on to expert reviewers, and only the accepted phrases are added to CIT_V_1.1_ to obtain CIT_V_1.2_. The rejected phrases are stored and are automatically excluded from consideration, should they be generated again during a later iteration.

### Consecutive iterations of concatenation and anchoring

**Fig. 6:**
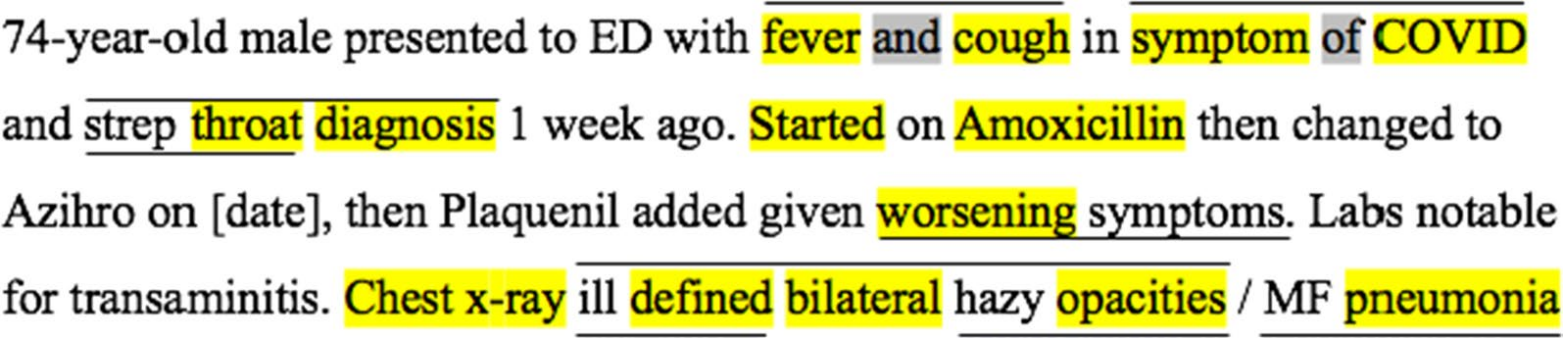
An excerpt from a synthetic EHR illustrates some example phrases obtained by consecutive concatenation and anchoring operations. Annotated words are highlighted in yellow and stop words contained in such phrases are highlighted in grey. Overbars represent concatenation and underlines represent anchoring

The CIT is enriched with fine granularity phrases by performing alternating iterations of concatenation and anchoring. We note that phrases obtained by concatenation, may be combined with unannotated words by performing anchoring in a successive iteration. Similarly, phrases obtained by anchoring may be concatenated in the next iteration to form new fine granularity phrases. Figure 5 illustrates an example of the latter case in which *peripheral consolidations* obtained by anchoring will, in the next iteration, be concatenated with “bilateral” to obtain *bilateral peripheral consolidations*. Another such example is *bilateral airspace opacification*. In Fig. 6, the concept *ill-defined bilateral hazy opacities* is obtained by concatenation of three phrases, two of which are obtained earlier by anchoring.

In fact, the only concepts added in consecutive iterations are those that combine concepts that were added in the previous iteration. The reason is that, if a phrase mined in a later iteration contains only concepts from CIT_V_0_, it should have already been mined in the first iteration of concatenation or anchoring. Such a concept was either already added to CIT_V_0_ upon review or was rejected. If it was rejected, then it is stored as a rejected phrase and will not be reconsidered.

After every iteration of concatenation and anchoring, we perform a check for convergence. The process of building the CIT is defined to converge when the sum of the number of accepted phrases obtained by concatenation and anchoring in one iteration falls below a given threshold value. In this study, we use a threshold of 50.

### Review process

The phrases obtained by concatenation/anchoring in each iteration were reviewed by three reviewers (VK, SZ, and LL). Each review proceeded through three rounds. The reviewers were provided with the list of phrases obtained by concatenation/anchoring along with the context (the sentence) in which each phrase appears. In the first round, each reviewer independently reviewed the phrases for inclusion/exclusion in CIT and submitted the final decisions to the review moderator (LZ). The moderator divided the phrases into three groups: (1) Phrases accepted by all reviewers (2) Phrases rejected by all reviewers and (3) Phrases with no consensus. In the second round, the reviewers were provided with all the phrases in group 3 where consensus was not achieved along with a decision summary (i.e., 2 yes, 1 no or 1 yes, 2 no) to re-evaluate their decisions (and possibly change them). The revised decisions were returned to the moderator.

The moderator performed another similar round of division into three groups. The phrases for which consensus was not achieved after the second round were reviewed again in the third round. In this final round, a face-to-face (F2F) discussion was conducted to resolve the differences. If at any of the rounds a reviewer marked a phrase as "maybe," the phrase was reviewed by domain experts (GE or AE who are MDs with experience with COVID cases). Phrases that obtained a "yes" consensus by the reviewers in any of the rounds or were approved by either GE or AE were added to the CIT. The moderator (LZ) calculated the interrater reliability [77] for the first two rounds of each review using the measures 'percent agreement' and 'Fleiss Kappa' to evaluate the agreement among the three reviewers.

With several iterations of concatenation and anchoring, some long phrases may be obtained. The reviewers followed the rule that a complex concept may be added to CIT if it represents fine granularity information corresponding to chunks [22, 78].

In addition to the accept/reject decision, the reviewers also identified multiple phrases that are synonyms during each iteration. These phrases were grouped and added as a single concept with one phrase representing the concept name and the others labeled as synonyms. It is also possible that newly accepted phrases are synonyms of already existing concepts in CIT. We use the same approach of string matching (both exact and fuzzy matching) to deal with this situation.

### Performance metrics

We define two performance metrics: *Coverage* is the percentage of words being annotated. *Breadth* is the average number of words per annotated concept.

2 $$Coverage = \frac{\# annotated\; words}{{\# all \,words}} \times 100$$

3 $$Breadth = \frac{\# annotated \;words}{{\# annotated\; concepts}}$$

The rationale for the coverage metric is that the higher the coverage is, the higher the amount of meaningful clinical information captured from the EHR will be. For example, the coverage of the excerpt in Fig. 3 is 7/27 = 26% with ICIT, and 12/27 = 44% with CIT_V_0_.

The rationale for the breadth metric is that longer phrases help to better convey a chunk describing clinical information. For example, the chunk *extensive bilateral pulmonary infection*, with breadth 4 appears in Fig. 4. It conveys the intended clinical information much better than its three constituting SNOMED CT concepts, with breadth value of 1.33. Thus, capturing chunks rather than discrete individual concepts increases breadth.

### Automatic enhancement of the annotation coverage for *DS*_*test*_

**Fig. 7:**
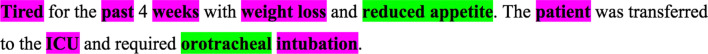
An excerpt from the Radiopaedia test case [21] of a COVID patient with concepts present in CIT_V_4.2_ (highlighted in pink) and DIFF' concepts from SNOMED CT (highlighted in green)

Since the CIT is enriched by phrases mined from *DS*_*build*_, it is natural that the annotation coverage of *DS*_*build*_ with CIT will be increased after several iterations. The question is what coverage will be achieved by annotating *DS*_*test*_ by the same version of the CIT. In Fig. 7, the pink highlights show the text of an excerpt from *DS*_*test*_ that is annotated with the CIT Version 4.2.

The annotated text in the figure contains SNOMED CT concepts (highlighted in green) e.g., *reduced appetite*. The reason why those SNOMED CT concepts were not yet added to the CIT is that they did not appear in *DS*_*build*_, because those SNOMED CT concepts that appear in *DS*_*build*_ were already identified and added to CIT_V_0_ in the beginning of the process. Therefore, we can perform the DIFF operation for automatically identifying these concepts and add them to CIT to obtain CIT'. In this way, we can increase the annotation coverage for *DS*_*test*_ by using CIT'.

4 $$DIFF^{\prime} = \, \left\{ {DS_{test} \;annotated \, \,with\; \, SNOMED \, \;CT} \right\} \, {-} \, \left\{ {DS_{test} \;annotated \, \;with \, \;CIT} \right\}$$

5 $$CIT^{\prime} = CIT \cup DIFF^{\prime }$$

For example, for the excerpt of Fig. 7, the coverage of *DS*_*test*_ with CIT is 8/23 = 35% but the coverage of *DS*_*test*_ with CIT' is 11/23 = 48%. This enhancement of CIT can be performed automatically and enables the adjustment of the interface terminology for each new dataset of clinical notes. In this way, CIT can be dynamically adjusted to new datasets.

## Results

As described in the Method Section, the CIT is curated in iterations. We performed alternating applications of the concatenation and anchoring operations. Hence, for V_i_, i = 1, 2, …, it is the case that V_i.1_ represents the version of CIT obtained by applying concatenation to the version V_i-1.2_ (that is, i-1). V_i.2_ represents the version of CIT obtained by applying anchoring to the version V_i.1._ For example, version V_2.1_ is obtained by applying concatenation to the version V_1.2_.

In Table 1, we described the review process of candidate phrases by the domain experts. After each operation, there are three stages of the review. In the table, for each stage, we list the number of phrases accepted as concepts into CIT, and their cumulative percentage, after all three reviewers accepted them. In the last three rows, the intermediate consensus stage was skipped, going directly to the F2F consensus stage, in view of the low number of phrases to be reviewed. Even after the F2F meeting, a decision was still not agreed on for a few phrases. For such cases, GE or AE made the final judgement. The phrases accepted by GE or AE were also added to CIT. Hence, the total number of accepted phrases is the summation of the accepted phrases from the first two stages of review, the F2F meeting, and the final resolution by GE.

**Table 1:** Statistics of extracted phrases for all versions

Version	Procedure	Total # of phrases extracted	# phrases accepted after 1st review	% phrases accepted after 1st review	# phrases accepted during 2nd review	% phrases accepted after two reviews	# phrases accepted after F2F meeting	Total # of phrases accepted	% retained w.r.t. total # phrases extracted
CIT_V_1.1_	Concatenation	1197	251	20.97%	125	31.41%	120	525	43.86%
CIT_V_1.2_	Anchoring	2488	663	26.65%	198	34.60%	52	937	37.66%
CIT_V_2.1_	Concatenation	972	449	46.19%	70	53.40%	33	556	57.20%
CIT_V_2.2_	Anchoring	1345	186	13.83%	41	16.88%	43	280	20.82%
CIT_V_3.1_	Concatenation	395	82	20.76%	42	31.39%	29	154	38.99%
CIT_V_3.2_	Anchoring	206	47	22.82%	–	–	18	66	32.04%
CIT_V_4.1_	Concatenation	101	9	8.91%	–	–	17	26	25.74%
CIT_V_4.2_	Anchoring	133	8	6.02%	–	–	1	9	6.77%

Table 2 reports the interrater reliability of the first two rounds of reviews among the three reviewers for each iteration measured by 'percent agreement' and 'Fleiss’ Kappa'. For example, for the iteration CIT_V_1.2_, in the first two rounds of the review, the percent agreement was 0.9 and 0.94, and the Fleiss’ Kappa was 0.6 and 0.78. Interestingly, the three reviewers have a higher agreement for the anchoring versions than that for the concatenation versions.

**Table 2:** The interrater reliability of the reviews among the three reviewers

Version	Procedure	Percent agreement for 1st review	Fleiss’ Kappa for 1st review	Percent agreement for 2nd review	Fleiss’ Kappa for 2nd review
CIT_V_1.1_	Concatenation	0.85	0.46	0.91	0.67
CIT_V_1.2_	Anchoring	0.90	0.60	0.94	0.78
CIT_V_2.1_	Concatenation	0.87	0.43	0.92	0.67
CIT_V_2.2_	Anchoring	0.91	0.54	0.94	0.72
CIT_V_3.1_	Concatenation	0.84	0.37	0.92	0.69
CIT_V_3.2_	Anchoring	0.92	0.66	–	–
CIT_V_4.1_	Concatenation	0.83	0.23	–	–
CIT_V_4.2_	Anchoring	0.96	0.58	–	–

In Table 3, we list examples of accepted concepts. In the top half of the table, we list those concepts created by concatenation where each concatenated concept is enclosed with ‘|’ to the left and right of the concept name. The rows 1, 3, 4 and 5 show the concatenation of three concepts. The last concatenation example contains a stop word. The bottom half of the table contains examples of anchoring. The anchor concepts are shown in bold font. The last example contains two stop words.

**Table 3:** Examples of accepted phrases from CIT_V_1_

Version	Accepted phrases
CIT_V_1.1_ (Concatenation)	|widespread| |bilateral| |consolidation|
	|microvascular| |dilation|
	|extensive| |lung| |damage|
	|left| |upper| |paratracheal lymphadenopathy|
	|diffuse| |bilateral| |consolidation|
	|opacification| in |both lungs|
CIT_V_1.2_ (Anchoring)	**atelectatic** bands
	**peripheral** predominance
	crazy-paving **pattern**
	**air** bronchograms
	subpleural **fibrous** streak
	airspace **opacification** in the lungs

In Table 4, we present examples of phrases rejected during the review process. The major cause for rejection of phrases in the initial stages was that the phrases generated are not complete. For example, *|cases| of |mild|* generated by concatenation (row 3) is a partial phrase (ending with an adjective and missing a noun to its right) corresponding to the complete phrase "*cases of mild disease.*" In the Discussion Section we describe in detail why this happens and how complete phrases, corresponding to these partial ones will be generated in the later iterations. We also show similar examples of incomplete phrases generated by anchoring (Table 4). For anchoring, since we use three rules for generating potential phrases, in some cases we obtain both partial and complete phrases in the same iteration. For example, the phrases *Congested ****central***, ***central**** vessels* and *Congested ****central**** vessels* are generated by using *central* as the anchor concept, of which the first phrase is partial and hence rejected (Table 4).

**Table 4:** Examples of rejected phrases

Procedure	Rejected phrases
Concatenation	|patient| had |positive|
	|small| |foci of|
	|cases| of |mild|
	|bilateral| |mid|
	|endotracheal tube| and |right|
Anchoring	**disease** in terms
	particularly at **lower**
	**SARS-CoV-2** and the typical
	Congested** central**
	**segments** of **both upper**
	arched **shape** and occupies

In Table 5, we show some examples of long phrases that were obtained by a sequence of concatenation and anchoring operations. Those phrases were accepted by the reviewers into the CIT as concepts, because they capture fine granularity information corresponding to chunks [22, 76]. Those phrases are complex, because they contain many adjectives and/or associated phrases further describing the main object. For example, in the first row, the main phrase is *ill-defined bilateral alveolar consolidation,* and the associated phrase is *with peripheral distribution.* The subject of this phrase is *alveolar consolidation*. It is described by the two adjectives preceding it and is further elaborated by the associated phrase.

**Table 5:** Examples of long accepted phrases

Long accepted phrases
|ill-defined| |bilateral| |**alveolar consolidation**| with |peripheral distribution|
|inter-|/ |intra-| |**lobular septal** thickening|
|bilateral| |consolidations with| |**air bronchogram**|
|rapidly| |progressive| |acute| |**respiratory distress syndrome**|
|calcified| |**mediastinal|** and |**hilar|** |enlarged| |lymph nodes|
|segmental| and |subsegmental| |confluent **acinar consolidation|**
|peripheral| |large| |areas of **ground glass**| with |small| |perivascular consolidations|
|discrete| |bilateral| |ground|-|glass| |**opacities|** with |round **morphology|**

In Table 6, we show the progression of the values of the coverage and the breadth metrics during the process of constructing the CIT. For annotating with CIDO alone, we obtained only 10.49% coverage for *DS*_*build*_, since the text of EHR notes of COVID patients did not have a high percentage of COVID-related terms. The breadth was 1.13, because most concepts consisted of one word. For the ICIT, which integrates six COVID ontologies and adds COVID related concepts from several general terminologies, 4202 concepts were added to the concepts of CIDO. This expansion approximately doubled the coverage to 21.36%.

**Table 6:** Coverage and breadth for *DS*_*build*_

Version	# concepts	Coverage	Breadth
CIDO	7834	10.49%	1.13
SNOMED_CT	354,178	46.94%	1.18
ICIT	12,036	21.36%	1.14
CIT_V_0_	12,697	53.73%	1.17
CIT_V_1.1_	13,156	55.51%	1.38
CIT_V_1.2_	13,970	61.75%	1.78
CIT_V_2.1_	14,166	62.33%	2.19
CIT_V_2.2_	14,430	66.34%	2.30
CIT_V_3.1_	14,594	66.82%	2.35
CIT_V_3.2_	14,658	67.43%	2.37
CIT_V_4.1_	14,686	67.50%	2.39
CIT_V_4.2_	14,686	67.59%	2.40

In contrast, with the large clinical terminology SNOMED CT, with 354,178 concepts, a coverage of 46.96% was obtained. The much higher coverage was obtained, because SNOMED CT contains medical concepts, e.g., medical conditions and medications that are not necessarily COVID related, but appear, for example, in the medical history of a patient. In addition, SNOMED CT also contains general English concepts used in EHR notes, e.g., time-related concepts. The breadth is only slightly higher (1.18), because most of the SNOMED CT concepts, which appear in *DS*_*build*_, consist of one word.

In CIT_V_0_, we added to ICIT the concepts of the DIFF (see (1)) between SNOMED CT and ICIT. Thus, all the 661 concepts of SNOMED CT that appear in *DS*_*build*_*,* but do not appear in ICIT were inserted into CIT_V_0_. As a result, the coverage of *DS*_*build*_ for the CIT_V_0_, at 53.73%, is higher than both individual coverages of SNOMED CT and ICIT.

With alternating applications of concatenation and anchoring, we achieved meaningful increases in the first two iterations. The increases in the two following iterations were low. During the fourth iteration, only 28 (< 50) concepts were added to CIT; thus, convergence was achieved, and the process stopped. The final coverage for *DS*_*build*_ is 67.59% and the breadth is 2.4.

### Results for *DS*_*test*_

As was seen above, the annotation with the final version of the CIT achieved a coverage of *DS*_*build*_ that was higher by 21% than the coverage of *DS*_*build*_ with SNOMED CT. The coverage obtained for *DS*_*test*_ was 11.23% for CIDO, 21.52% for ICIT and 46.74% for SNOMED CT. For those three terminologies, the coverages for *DS*_*build*_ and *DS*_*test*_ are similar, since those three terminologies do not depend on *DS*_*build*_. Interestingly, the results are close for CIT_V_0_, where the coverage is 53.33% for *DS*_*test*_ vs 53.73% for *DS*_*build*_, even though the DIFF was extracted from *DS*_*build*_ and may have missed SNOMED CT concepts that appeared only in *DS*_*test*_. Apparently, most of the concepts of SNOMED CT in *DS*_*test*_ were also in *DS*_*build*_.

Using (4) and (5) in the Method section, we calculated DIFF' based on *DS*_*test*_ and created CIT' = CIT_V_4.2_ ∪ DIFF'. The final annotation for *DS*_*test*_ was performed by CIT' obtaining a coverage of 59.46%, and a breadth of 1.68. In comparison, for *DS*_*build*_, the final CIT obtained a coverage of 67.59% and a breadth of 2.4. Hence, our technique achieved for *DS*_*test*_ a coverage that is about 88% of the coverage obtained for *DS*_*build*_. Even though CIT was built by extracting concepts from *DS*_*build*_, nevertheless, it was quite effective for annotation of *DS*_*test*_ with CIT'. CIT' was obtained by enriching CIT with DIFF'.

**Fig. 8:**
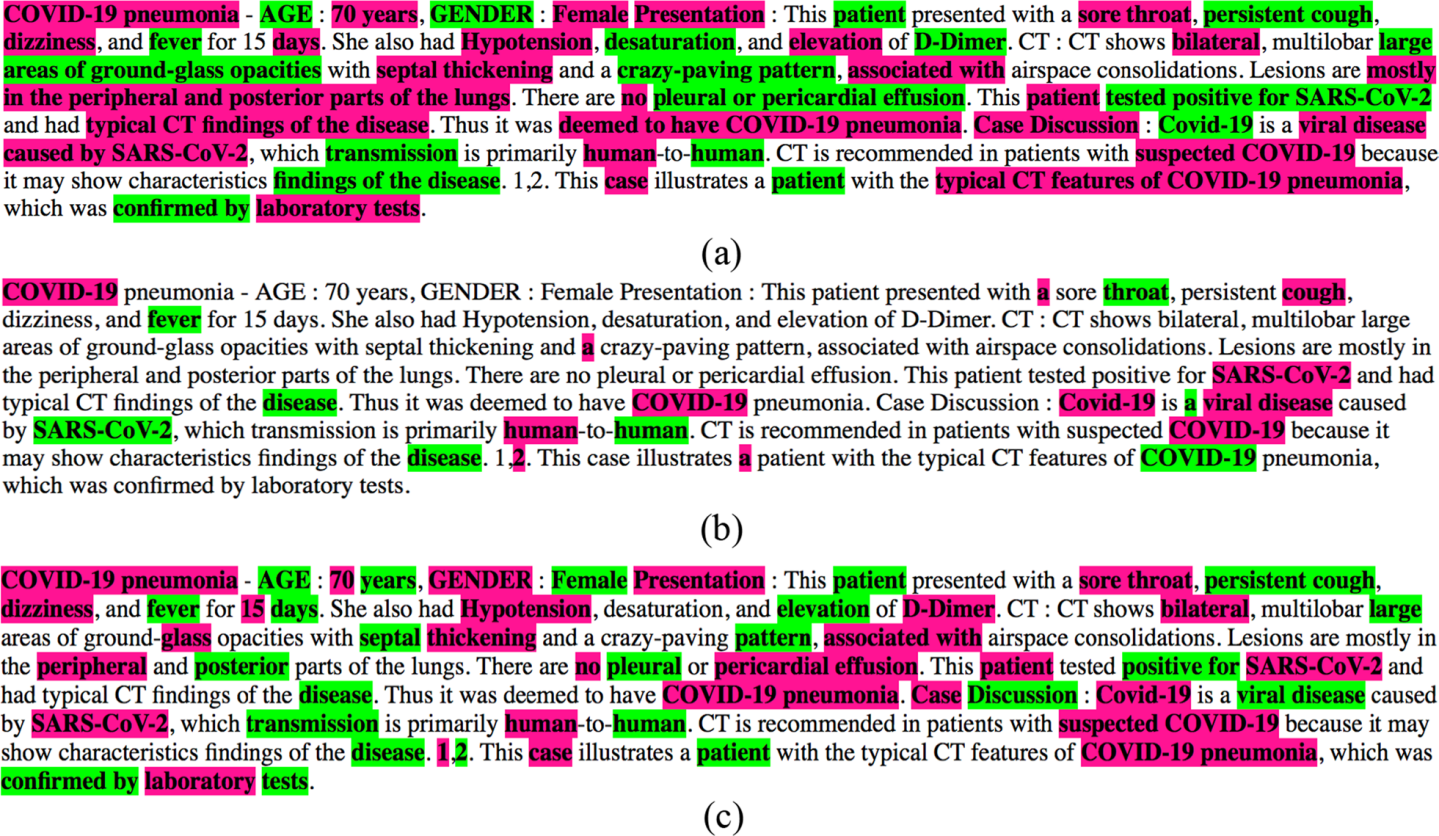
An example clinical note annotated by **a** CIT_V_4.2’_, **b** CIDO, and **c** SNOMED CT. Annotated phrases are highlighted alternatingly with pink and green colors to better distinguish between consecutive annotated phrases

To illustrate the capacity of an annotated text to capture the content of an EHR note, we present a note from the test dataset with annotation coverage level which is close to the average annotation coverage obtained for the test dataset with the CIT_V_4.2’_. Furthermore, to emphasize the difference in the capability of various terminologies to capture the content of the clinical note we show in Fig. 8 the annotation of the same note with CIT_V_4.2’_, CIDO and SNOMED CT in parts (a), (b) and (c), respectively. The reader can try to read only the annotated text and assess to what extent it is reflecting the content of the clinical note. The figure uses the pink and green colors alternatingly to mark the annotated phrases to easily distinguish between consecutive phrases.

In part (b) showing the annotation with CIDO, only a few, most common concepts related to COVID-19 are annotated. The annotated text fails to communicate the content of the note. The situation in part (c) is much better. A meaningful portion of the content is captured with the SNOMED CT concepts, but some parts of the text, especially parts related to the CT images are not captured. The annotation is still not capturing the complete content in a satisfactory fashion. In contrast in part (a) which shows annotation with CIT_V_4.2’_ a substantial portion of the content of the clinical notes is captured in the annotated phrases. Numerically this is manifested by the difference in the coverage metrics, 60.53% versus 41.45%. We consider some examples: "large areas of ground-glass opacities" is an annotated concept of CIT_V_4.2’_ capturing the chunk by which a radiologist describes a CT image typical for COVID patients. In contrast the SNOMED CT annotation captures only two isolated words "large" and "glass" of this chunk. Another example is "crazy paving pattern" which is a concept of CIT_V_4.2’_, but only "pattern" is annotated for SNOMED CT from this chunk. At the same time, we acknowledge many phrases that are captured by SNOMED CT e.g., "suspected COVID-19" and "COVID-19 pneumonia".

Another distinction between the annotation with CIT_V_4.2’_ and with SNOMED CT is that CIT_V_4.2’_ has concepts which are long chunks missing in SNOMED CT. This difference is manifested by the metric 'Breadth' with 2.24 for CIT_V_4.2’_ versus 1.21 for SNOMED CT. Consider for example the chunk "mostly in the peripheral and posterior parts of the lungs" which is a concept in CIT_V_4.2’_. In contrast, only two words "peripheral" and "posterior" are annotated separately for SNOMED CT. Such chunks with their semantics are necessary for capturing the content of clinical notes for performing automated research on textual data and many such chunks are missing in reference terminologies which can be attributed to their design principles.

## Discussion

When we compare the first applications of concatenation and anchoring (see Table 1), 2488 phrases were obtained by anchoring, while only 1197 phrases were obtained by concatenation. The reason for this is that in anchoring, at most three phrases can be created for each CIT concept annotated in the text, one with the word to the left, one with the word to the right and the final one with words to the left and right. In contrast, for concatenation, two or more adjacent concepts annotated in the text are required to obtain one fine granularity phrase. Thus, there is more potential for anchored phrases than for concatenated phrases. The same phenomenon occurs in the second round of concatenation and anchoring. In later rounds, there are fewer, longer phrases and the situation becomes more complex.

The total numbers of extracted phrases for concatenation in each iteration decrease monotonically: 1197, 972, 395 and 102. We note that the phrases created in the second round of concatenation contain at least one new concept created during the previous anchoring iteration, since otherwise they would have already been created and evaluated in the first concatenation iteration. (Note that once a concept is evaluated and rejected, it is automatically removed from consideration if it is created again, as mentioned in the Method Section.) Since new phrases created by concatenation depend on the number of phrases accepted in the previous anchoring iteration, a lower number of phrases in the second round is expected. A concatenated phrase created in the third round must similarly contain a concept obtained in the second anchoring iteration and so on, decreasing the number of phrases obtained in each round.

Correspondingly, a phrase obtained by anchoring in the second iteration, is created by anchoring a new concept accepted in the previous concatenation or anchoring iterations. Otherwise, such a phrase would have been created in the first anchoring iteration. There is, again, a monotonous decrease in the numbers of extracted phrases during the anchoring iterations, with 2055, 1345, 206 and 134 phrases, respectively. The rationale for this monotonicity is similar to the situation for concatenation, even though an anchored new concept could have been created either in the previous concatenation or in the previous anchoring iteration. Note that we can concatenate more than two concepts, but anchoring is limited to adding only one unannotated word (with zero or more stop words) to an annotated concept.

Since a phrase created in any iteration after the first one must contain concepts created in the previous iteration, phrases become longer as we advance in the process. Longer phrases have lower likelihoods to be accepted by the expert reviewers. Thus, the final percentage of accepted phrases decreases with every concatenation iteration.

In Table 3, we have shown some examples of phrases that were not accepted for inclusion in the CIT by the reviewers. In the Results Section, we analyzed some incomplete phrases and their corresponding complete phrases. For example, the phrase “*cases of mild disease”* is obtained by concatenating three concepts, *cases*, *mild,* and *disease* together with the stop word *of*. What matters here is the order in which the individual concepts are combined. While “*cases of mild*” is a partial phrase (and hence rejected), “*mild disease*” and “*cases of mild disease”* are potential candidates for inclusion. Thus, even though “*cases of mild*” is rejected we can still obtain “*cases of mild disease”* in a future iteration by first accepting “*mild disease*” and then concatenating it with the concept *cases*. There is no loss of information except that the generation of such phrases is delayed enabling the correct order of combination.

Another example is the phrase “*particularly at lower lobes.*” First, we create |lower**| |**lobes**|** by concatenation and in the next anchoring iteration, the anchor will be ***lower lobes*** and “*particularly at lower lobes*” will be obtained. Those examples demonstrate that the complete phrases corresponding to incomplete phrases that were rejected by the reviewers, will be generated in an alternative way, if they are potential candidates for inclusion in the CIT.

In Table 5, we showed the coverage and the breadth for each iteration. We observe that the anchoring iterations display higher increases in the coverage than the concatenation iterations. The reason for this is that, in concatenation, we combine two adjacent concepts in the text into a single concept, but not much gain is achieved for the coverage besides the stop words that become parts of the concatenated phrases. For anchoring, since unannotated words are combined with annotated concepts the coverage increases significantly. On the other hand, the breadth increases during concatenation, as multiple concepts are combined into one and the number of words per concept increases.

With regards to the *DS*_*test*_ result, we note that the breadth for *DS*_*test*_ annotated by CIT' is 1.68 vs. 2.40 for *DS*_*build*_ with CIT_V_4.2_. The reason is that DIFF', which contains concepts of SNOMED CT that were not included in CIT_V_4.2,_ consists of short SNOMED CT concepts in contrast to the longer concepts of CIT created by concatenation and anchoring operations. In previous research [29], we calculated the coverage only for *DS*_*build*_, since we did not create a *DS*_*test*_ dataset. It is encouraging to see, in the current research, that the gap between the coverages for *DS*_*test*_ and *DS*_*build*_ is relatively small i.e., the coverage for *DS*_*test*_ is about 88% of the coverage for *DS*_*build*_.

This paper describes Phase 1 of a research project to construct the CIT for annotation of EHR notes of COVID patients. In the future second phase, the concepts that were added during the iterations of concatenation and anchoring will serve as training data for advanced machine learning techniques. Using deep-learning models such as BioSyn [79] and DILBERT [80] will enable us to achieve better concept normalization as these models can identify most semantically similar terms corresponding to concepts in a terminology and provide better embeddings.

As we see in Table 2, the interrater reliability among the three reviewers in the first iteration for the phrases obtained by both concatenation and anchoring operations is low. The reason for this is that the decision whether a phrase obtained by concatenation or anchoring fits to be in CIT is often a difficult decision. On one hand, there are easy cases where a phrase is obviously a partial phrase (as explained previously) and will be rejected by all three reviewers, and easy cases where a phrase is clearly fitting as a concept of CIT and all three reviewers are likely to accept. On the other hand, for many phrases there is no clear-cut decision, especially when the phrases become longer.

Even in case of reference terminologies, curators sometimes disagree whether a concept should be added to the terminology. The decision for an interface terminology is even harder. There is no "definition" for the qualities justifying which phrases deserve to be inserted into an interface terminology, beyond being a concept and serving the purpose of the application for which the interface terminology is created. Many times, a reviewer is debating options before deciding. Thus, it is not surprising that interrater reliability is low. Nevertheless, the agreement improves in the 2nd review, after each reviewer has a chance to see the anonymous decisions of the other reviewers. In the third review, which is face to face interaction, the three reviewers were able to come to a consensus decision for almost all leftover phrases. Remaining phrases were decided by (GE) or (AE).

This situation is similar to another problem which the SABOC team encountered in the past. Each concept of the UMLS Metathesaurus [14] is assigned one or more Semantic Type (ST) out of 131 STs of the UMLS Semantic Network [81]. The problem was Quality assurance of the Semantic Type (ST) assignments of UMLS concepts which were assigned multiple semantic types [82–90]. While deciding the ST of a regular UMLS concept is typically straightforward, some concepts have a multifaceted nature and deserve multiple STs. Such concepts are typically more complex and the decision about their proper ST assignment is more difficult. We have shown that for such cases the error rate of ST assignments was much higher than for concepts with only one assigned ST. The SABOC team reported many such errors to the National Library of Medicine and most of them were indeed corrected in following releases of the UMLS. Similar to our current study, we saw many cases of disagreement among reviewers. A study [91] on the reliability of four reviewers who were all well versed with the UMLS found that each one of the reviewers was not reliable but that their consensus was reliable. Acknowledging that we are dealing in the current study with difficult decisions for some phrases, we enrolled three reviewers in our study and conducted three rounds of reviews for each iteration including a final face-to-face review.

A natural question that arises is whether the concepts in CIT can be integrated into existing ontologies such as SNOMED CT to enrich their conceptual content. While ideally integrating CIT concepts into existing ontologies is useful, there exist some concerns regarding the feasibility. The main concern is whether the fine granularity concepts in CIT would be a fit for inclusion in reference terminologies. Every ontology/terminology is based on some design principles that are rarely made available to researchers outside of the organization. Hence, the final decision concerning integration, needs to be made by curators of the ontology, who has to balance factors of completeness against efficiency, usability, and maintainability. In addition, terminologies such as SNOMED CT do not have a large number of basic COVID-19 concepts, so adding fine granularity concepts would require extra work in terms of restructuring the hierarchies and including additional concepts that were incorporated into CIT from CIDO and other COVID-related ontologies. For large ontologies/terminologies, this is not an easy task.

A more feasible option for integration would be to consider ontologies such as CIDO, that are specifically designed for COVID-related concepts. Since we have worked with curators of CIDO [56] and considering that CIDO is continuously maintained, in the future we will explore plans to make CIT available to curators of CIDO for concept enrichment.

### Limitations

This research was done with a relatively small dataset. We expect higher coverage when a larger dataset will be used, because for a larger dataset, CIT will capture more common phrases used in EHR notes of COVID patients. Furthermore, we expect the gap between the coverages of *DS*_*build*_ and *DS*_*test*_ to become smaller for a larger dataset. In future research, we will utilize a larger dataset of EHR notes and will also make CIT publicly available for peer reviews.

In the current research, we used manual review of the phrases mined from the EHR notes using concatenation and anchoring operations. In future, we plan to reduce the time for the review process by automatically rejecting partial phrases. For example, using part-of-speech taggers [92, 93] we can identify phrases that end with an adjective (e.g., *cases of mild*). In addition, key phrase extraction algorithms will be utilized to automatically extract characteristic and representative phrases from clinical notes [94, 95].

## Conclusions

In this research, we constructed a COVID-19 Interface Terminology for the purpose of annotating EHR notes of COVID patients. The CIT developed by integrating concepts from existing ontologies and mining concepts from EHR notes, contains fine granularity concepts and is rich in synonyms. The CIT incorporating the collection of concepts added to it in this first phase of research, will serve as training data for machine learning techniques in the second phase, thereby enhancing the CIT even more and scaling the annotations to larger volumes of EHR notes of COVID-19 patients.

## Data Availability

The case studies supporting the conclusions of this article are available on Radiopaedia at https://radiopaedia.org/search?utf8=%E2%9C%93&q=covid-19&scope=all&lang=us. The build and test splits of the dataset used in our study is available at https://github.com/ericaSS/Radiopeadia_Covid_Data/tree/main/data.

## Abbreviations

CIT: COVID Interface Terminology
CIDO: Coronavirus Infectious Disease Ontology
EHRs: Electronic Health Records
UMLS: Unified Medical Language System
MedLEE: Medical Language Extraction and Encoding
ICIT: Initial COVID Interface Terminology
CPT: Current Procedural Terminology
ICD: International Classification of Diseases
LOINC: Logical Observation Identifiers, Names and Codes
NDC: National Drug Code

## References

[1] Uzuner O, South BR, Shen S, DuVall SL. 2010 i2b2/VA challenge on concepts, assertions, and relations in clinical text. J Am Med Inform Assoc. 2011;18:552–6. 21685143 10.1136/amiajnl-2011-000203 PMC3168320

[2] Henry S, Buchan K, Filannino M, Stubbs A, Uzuner O. 2018 n2c2 shared task on adverse drug events and medication extraction in electronic health records. J Am Med Inform Assoc. 2020;27:3–12. 31584655 10.1093/jamia/ocz166 PMC7489085

[3] Shickel B, Tighe PJ, Bihorac A, Rashidi P. Deep EHR: a survey of recent advances in deep learning techniques for electronic health record (EHR) analysis. IEEE J Biomed Health Inform. 2018;22:1589–604. 29989977 10.1109/JBHI.2017.2767063 PMC6043423

[4] Datta S, Bernstam EV, Roberts K. A frame semantic overview of NLP-based information extraction for cancer-related EHR notes. J Biomed Inform. 2019;100:103301. 31589927 10.1016/j.jbi.2019.103301 PMC11771512

[5] Sun W, Rumshisky A, Uzuner O. Evaluating temporal relations in clinical text: 2012 i2b2 Challenge. J Am Med Inform Assoc. 2013;20:806–13. 23564629 10.1136/amiajnl-2013-001628 PMC3756273

[6] Pradhan S, Elhadad N, Chapman WW, Manandhar S, Savova GK. SemEval-2014 Task 7: analysis of clinical text. *SEMEVAL2014.

[7] Luo Y, Thompson WK, Herr TM, Zeng Z, Berendsen MA, Jonnalagadda SR, et al. Natural language processing for ehr-based pharmacovigilance: a structured review. Drug Saf. 2017;40:1075–89. 28643174 10.1007/s40264-017-0558-6

[8] Ohno-Machado L. Realizing the full potential of electronic health records: the role of natural language processing. J Am Med Inform Assoc. 2011;18:539. 21846784 10.1136/amiajnl-2011-000501 PMC3168331

[9] Wang X, Hripcsak G, Markatou M, Friedman C. Active computerized pharmacovigilance using natural language processing, statistics, and electronic health records: a feasibility study. J Am Med Inform Assoc. 2009;16:328–37. 19261932 10.1197/jamia.M3028 PMC2732239

[10] Chen J, Druhl E, Polepalli Ramesh B, Houston TK, Brandt CA, Zulman DM, et al. A natural language processing system that links medical terms in electronic health record notes to lay definitions: system development using physician reviews. J Med Internet Res. 2018;20:e26. 29358159 10.2196/jmir.8669 PMC5799720

[11] Jonquet C, Shah NH, Musen MA. The open biomedical annotator. Summit Transl Bioinform. 2009;2009:56–60. 21347171 PMC3041576

[12] Aronson AR, Lang FM. An overview of MetaMap: historical perspective and recent advances. J Am Med Inform Assoc. 2010;17:229–36. 20442139 10.1136/jamia.2009.002733 PMC2995713

[13] Savova GK, Masanz JJ, Ogren PV, Zheng J, Sohn S, Kipper-Schuler KC, et al. Mayo clinical text analysis and knowledge extraction system (cTAKES): architecture, component evaluation and applications. J Am Med Inform Assoc. 2010;17:507–13. 20819853 10.1136/jamia.2009.001560 PMC2995668

[14] Bodenreider O. The unified medical language system (UMLS): integrating biomedical terminology. Nucleic Acids Res. 2004;32:D267–70. 14681409 10.1093/nar/gkh061 PMC308795

[15] Donnelly K. SNOMED-CT: the advanced terminology and coding system for eHealth. Stud Health Technol Inform. 2006;121:279–90. 17095826

[16] He Y, Yu H, Ong E, Wang Y, Liu Y, Huffman A, et al. CIDO, a community-based ontology for coronavirus disease knowledge and data integration, sharing, and analysis. Sci Data. 2020;7:181. 32533075 10.1038/s41597-020-0523-6 PMC7293349

[17] Kanne JP, Bai H, Bernheim A, Chung M, Haramati LB, Kallmes DF, et al. COVID-19 imaging: what we know now and what remains unknown. Radiology. 2021;299:E262–79. 33560192 10.1148/radiol.2021204522 PMC7879709

[18] Kaufman AE, Naidu S, Ramachandran S, Kaufman DS, Fayad ZA, Mani V. Review of radiographic findings in COVID-19. World J Radiol. 2020;12:142–55. 32913561 10.4329/wjr.v12.i8.142 PMC7457163

[19] Rousan LA, Elobeid E, Karrar M, Khader Y. Chest x-ray findings and temporal lung changes in patients with COVID-19 pneumonia. BMC Pulm Med. 2020;20:245. 32933519 10.1186/s12890-020-01286-5 PMC7491017

[20] Mathy F, Feldman J. What’s magic about magic numbers? Chunking and data compression in short-term memory. Cognition. 2012;122:346–62. 22176752 10.1016/j.cognition.2011.11.003

[21] Tulving E, Patkau JE. Concurrent effects of contextual constraint and word frequency on immediate recall and learning of verbal material. Can J Psychol. 1962;16:83–95. 13923055 10.1037/h0083231

[22] Gobet F, Lane PC, Croker S, Cheng PC, Jones G, Oliver I, et al. Chunking mechanisms in human learning. Trends Cogn Sci. 2001;5:236–43. 11390294 10.1016/S1364-6613(00)01662-4

[23] Demner-Fushman D, Rogers WJ, Aronson AR. MetaMap Lite: an evaluation of a new Java implementation of MetaMap. J Am Med Inform Assoc. 2017;24:841–4. 28130331 10.1093/jamia/ocw177 PMC6080672

[24] SNOMED CT Compositional Grammer Specification and Guide. https://confluence.ihtsdotools.org/display/DOCSCG (accessed June 15th, 2020). 2021.

[25] Spackman KA, Campbell KE. Compositional concept representation using SNOMED: towards further convergence of clinical terminologies. Proc AMIA Symp. 1998;740–4. PMC2232179 9929317

[26] Minarro-Gimenez JA, Martinez-Costa C, Lopez-Garcia P, Schulz S. Building SNOMED CT post-coordinated expressions from annotation groups. Stud Health Technol Inform. 2017;235:446–50. 28423832

[27] Radiopeadia. https://radiopaedia.org/ (accessed Jun 15th, 2020). 2020.

[28] COVID-19 Database. https://www.sirm.org/category/senza-categoria/covid-19/ (accessed Nov 15th, 2019). 2021.

[29] Keloth V, Zhou S, Lindemann L, Elhanan G, Einstein A, Geller J, et al. Mining Concepts for a COVID Interface Terminology for Annotation of EHRs. In: 2020 IEEE International Conference on Big Data (Big Data). 2020;3753–60.

[30] Wang L, Foer D, MacPhaul E, Lo Y-C, Bates D, Zhou L. PASCLex: a comprehensive post-acute sequelae of COVID-19 (PASC) symptom lexicon derived from electronic health record clinical notes. J Biomed Inform. 2021. 10.1016/j.jbi.2021.103951 PMC8590503 34785382

[31] Zhou L, Plasek JM, Mahoney LM, Karipineni N, Chang F, Yan X, et al. Using medical text extraction, reasoning and mapping system (MTERMS) to process medication information in outpatient clinical notes. AMIA Annu Symp Proc. 2011;2011:1639–48. 22195230 PMC3243163

[32] Friedman C, Hripcsak G, DuMouchel W, Johnson SB, Clayton PD. Natural language processing in an operational clinical information system. Nat Lang Eng. 1995;1:83–108. 10.1017/S1351324900000061

[33] Health Information Text Extraction (HITEx). https://www.i2b2.org/software/projects/hitex/hitex_manual.html (accessed Jan 10th, 2020). 2006.

[34] Soldaini L. QuickUMLS: a fast, unsupervised approach for medical concept extraction. 2016.

[35] Noy NF, Shah NH, Whetzel PL, Dai B, Dorf M, Griffith N, et al. BioPortal: ontologies and integrated data resources at the click of a mouse. Nucleic Acids Res. 2009;37:W170–3. 19483092 10.1093/nar/gkp440 PMC2703982

[36] Wei CH, Allot A, Leaman R, Lu Z. PubTator central: automated concept annotation for biomedical full text articles. Nucleic Acids Res. 2019;47:W587–93. 31114887 10.1093/nar/gkz389 PMC6602571

[37] Kim D, Lee J, So CH, Jeon H, Jeong M, Choi Y, et al. A neural named entity recognition and multi-type normalization tool for biomedical text mining. IEEE Access. 2019;7:73729–40. 10.1109/ACCESS.2019.2920708

[38] Soysal E, Wang J, Jiang M, Wu Y, Pakhomov S, Liu H, et al. CLAMP - a toolkit for efficiently building customized clinical natural language processing pipelines. J Am Med Inform Assoc. 2018;25:331–6. 29186491 10.1093/jamia/ocx132 PMC7378877

[39] Kanter AS, Wang AY, Masarie FE, Naeymi-Rad FF, Safran C. Interface terminologies: bridging the gap between theory and reality for Africa. Stud Health Technol Inform. 2008;136:27–32. 18487703

[40] Zemmouchi-Ghomari L, Ghomari AR. Ontology versus terminology, from the perspective of ontologists. Int J Web Sci. 2012;1:315–31. 10.1504/IJWS.2012.052531

[41] Grabar N, Hamon T, Bodenreider O. Ontologies and terminologies: continuum or dichotomy? Appl Ontol. 2012;7:375–86. 10.3233/AO-2012-0119

[42] Schulz S, Jansen L. Formal ontologies in biomedical knowledge representation. Yearb Med Inform. 2013;8:132–46. 23974561

[43] Rosenbloom ST, Miller RA, Johnson KB, Elkin PL, Brown SH. Interface terminologies: facilitating direct entry of clinical data into electronic health record systems. J Am Med Inform Assoc. 2006;13:277–88. 16501181 10.1197/jamia.M1957 PMC1513664

[44] Rosenbloom ST, Brown SH, Froehling D, Bauer BA, Wahner-Roedler DL, Gregg WM, et al. Using SNOMED CT to represent two interface terminologies. J Am Med Inform Assoc. 2009;16:81–8. 18952944 10.1197/jamia.M2694 PMC2605600

[45] Wade G, Rosenbloom ST. Experiences mapping a legacy interface terminology to SNOMED CT. BMC Med Inform Decis Mak. 2008;8(Suppl 1):S3. 19007440 10.1186/1472-6947-8-S1-S3 PMC2582790

[46] Wade G, Rosenbloom ST. The impact of SNOMED CT revisions on a mapped interface terminology: terminology development and implementation issues. J Biomed Inform. 2009;42:490–3. 19285570 10.1016/j.jbi.2009.03.004 PMC2693286

[47] Rosenbloom ST, Miller RA, Johnson KB, Elkin PL, Brown SH. A model for evaluating interface terminologies. J Am Med Inform Assoc. 2008;15:65–76. 17947616 10.1197/jamia.M2506 PMC2274877

[48] Rosenbloom ST, Miller RA, Adams P, Madani S, Khan N, Shultz EK. Implementing an interface terminology for structured clinical documentation. J Am Med Inform Assoc. 2013;20:e178–82. 23384817 10.1136/amiajnl-2012-001384 PMC3715351

[49] BioPortal webpage of CIDO. https://bioportal.bioontology.org/ontologies/CIDO (accessed Dec 20th, 2020). 2008.

[50] Smith B, Ashburner M, Rosse C, Bard J, Bug W, Ceusters W, et al. The OBO Foundry: coordinated evolution of ontologies to support biomedical data integration. Nat Biotechnol. 2007;25:1251–5. 17989687 10.1038/nbt1346 PMC2814061

[51] Degtyarenko K, de Matos P, Ennis M, Hastings J, Zbinden M, McNaught A, et al. ChEBI: a database and ontology for chemical entities of biological interest. Nucleic Acids Res. 2008;36:D344–50. 17932057 10.1093/nar/gkm791 PMC2238832

[52] Elhanan G, Ochs C, Mejino JLV, Liu H, Mungall CJ, Perl Y. From SNOMED CT to Uberon: transferability of evaluation methodology between similarly structured ontologies. Artif Intell Med. 2017;79:9–14. 28532962 10.1016/j.artmed.2017.05.002 PMC5559337

[53] Ochs C, Perl Y, Halper M, Geller J, Lomax J. Quality assurance of the gene ontology using abstraction networks. J Bioinform Comput Biol. 2016;14(3):1642001. 27301779 10.1142/S0219720016420014

[54] Zheng L, Yumak H, Chen L, Ochs C, Geller J, Kapusnik-Uner J, et al. Quality assurance of chemical ingredient classification for the national drug file-reference terminology. J Biomed Inform. 2017;73:30–42. 28723580 10.1016/j.jbi.2017.07.013 PMC5652327

[55] Robinson PN, Kohler S, Bauer S, Seelow D, Horn D, Mundlos S. The Human phenotype ontology: a tool for annotating and analyzing human hereditary disease. Am J Hum Genet. 2008;83:610–5. 18950739 10.1016/j.ajhg.2008.09.017 PMC2668030

[56] Zheng L, Perl Y, He YO, Ochs C, Geller J, Liu H, et al. Visual comprehension and orientation into the COVID-19 CIDO ontology. J Biomed Inform. 2021;120:103861. 34224898 10.1016/j.jbi.2021.103861 PMC8252699

[57] COVID-19 Ontology. http://bioportal.bioontology.org/ontologies/COVID-19 (accessed Sept 30, 2020). 2020

[58] Sargsyan A, Kodamullil AT, Baksi S, Darms J, Madan S, Gebel S, et al. The COVID-19 ontology. Bioinformatics. 2020;36(24):5703–5. 10.1093/bioinformatics/btaa1057 PMC7799333 33346828

[59] Babcock S, Beverley J, Cowell LG, Smith B. The infectious disease ontology in the age of COVID-19. J Biomed Semant. 2021;12:13. 10.1186/s13326-021-00245-1 PMC8286442 34275487

[60] WHO COVID-19 rapid version CRF semantic data model. https://bioportal.bioontology.org/ontologies/COVIDCRFRAPID (accessed Sept 30, 2020). 2020.

[61] Infectious Disease Ontology. https://bioportal.bioontology.org/ontologies/IDO (accessed Sept 30, 2020). 2020.

[62] Virus Infectious Disease Ontology. https://bioportal.bioontology.org/ontologies/VIDO (accessed Sept 30, 2020). 2020.

[63] de Lusignan S, Lopez Bernal J, Zambon M, Akinyemi O, Amirthalingam G, Andrews N, et al. Emergence of a novel coronavirus (COVID-19): protocol for extending surveillance used by the royal college of general practitioners research and surveillance centre and public health England. JMIR Public Health Surveill. 2020;6:e18606. 32240095 10.2196/18606 PMC7124955

[64] Dutta B, DeBellis M. CODO: an ontology for collection and analysis of Covid-19 data. ArXiv. 2020;abs/2009.01210.

[65] ACT COVID Ontology v3.0. https://github.com/shyamvis/ACT-COVID-Ontology/tree/master/ontology (accessed Sept 30, 2020). 2020.

[66] WHO. International Classification of Diseases. http://www.who.int/classifications/icd/en/ (accessed Sept 30, 2020). 2020.

[67] McDonald CJ, Huff SM, Suico JG, Hill G, Leavelle D, Aller R, et al. LOINC, a universal standard for identifying laboratory observations: a 5-year update. Clin Chem. 2003;49:624–33. 12651816 10.1373/49.4.624

[68] Hirsch JA, Leslie-Mazwi TM, Nicola GN, Barr RM, Bello JA, Donovan WD, et al. Current procedural terminology; a primer. J Neurointerv Surg. 2015;7:309–12. 24589819 10.1136/neurintsurg-2014-011156

[69] National Drug Code Database Background Information. https://www.fda.gov/drugs/development-approval-process-drugs/national-drug-code-database-background-information (accessed Sept 30, 2020). 2017.

[70] Wang LL, Lo K, Chandrasekhar Y, Reas R, Yang J, Eide D, et al. CORD-19: the COVID-19 open research dataset. ArXiv. 2020.

[71] Global literature on coronavirus disease. https://search.bvsalud.org/global-literature-on-novel-coronavirus-2019-ncov/ (accessed Jun 15th, 2021). 2021.

[72] Sun Y, Butler A, Stewart LA, Liu H, Yuan C, Southard CT, Kim JH, Weng C. Building an OMOP common data model-compliant annotated corpus for COVID-19 clinical trials. J Biomed Inform. 2021;1(118):103790. 10.1016/j.jbi.2021.103790 PMC8079156 33887457

[73] Lee J, Kim JH, Liu C, Hripcsak G, Ta C, Weng C. COHD-COVID: Columbia Open Health Data for COVID-19 Research. medRxiv. 2020. 10.2196/31122 PMC8485985 34543225

[74] Lybarger K, Ostendorf M, Thompson M, Yetisgen M. Extracting COVID-19 diagnoses and symptoms from clinical text: a new annotated corpus and neural event extraction framework. ArXiv. 2021. 10.1016/j.jbi.2021.103761 PMC7997694 33781918

[75] Centers for Disease Control and Prevention. https://www.cdc.gov/coronavirus/2019-ncov/need-extra-precautions/people-with-medical-conditions.http (accessed Jun 1st, 2021). 2020.

[76] Daintith J. Kleene star. A dictionary of computing. 6th edN. Oxford University Press; 2008.

[77] McHugh ML. Interrater reliability: the kappa statistic. Biochem Med (Zagreb). 2012;22:276–82. 23092060 10.11613/BM.2012.031 PMC3900052

[78] Miller GA. The magical number seven plus or minus two: some limits on our capacity for processing information. Psychol Rev. 1956;63(2):81–97. 13310704 10.1037/h0043158

[79] Sung M, Jeon H, Lee J, Kang J. Biomedical entity representations with synonym marginalization. arXiv 2020. arXiv preprint arXiv:2005.00239. 2021.

[80] Miftahutdinov Z, Kadurin A, Kudrin R, Tutubalina E. Medical concept normalization in clinical trials with drug and disease representation learning. Bioinformatics. 2021;37(21):3856–64. 34213526 10.1093/bioinformatics/btab474 PMC8570806

[81] McCray A. The UMLS semantic network proceedings. In: Symposium on Computer Applications in Medical Care. 1989;503–507. PMCID: PMC2245676.

[82] Peng Y, Halper MH, Perl Y, Geller J. Auditing the UMLS for redundant classifications. In: Proceedings of AMIA Symposium. 2002; 612–6. PMID: 12463896; PMCID: PMC2244162. PMC2244162 12463896

[83] Chen Y, Gu HH, Perl Y, Geller J. Structural group-based auditing of missing hierarchical relationships in UMLS. J Biomed Inform. 2009;42(3):452–67. 18824248 10.1016/j.jbi.2008.08.006 PMC2714188

[84] Gu HH, Hripcsak G, Chen Y, Morrey CP, Elhanan G, Cimino JJ, Geller J, Perl Y. Evaluation of a UMLS auditing process of semantic type assignments. AMIA Ann Symp Proc. 2007;2007:294. PMC2655790 18693845

[85] Chen Y, Gu H, Perl Y, Halper M, Xu J. Expanding the extent of a UMLS semantic type via group neighborhood auditing. J Am Med Inform Assoc. 2009;16(5):746–57. 19567802 10.1197/jamia.M2951 PMC2744725

[86] Geller J, He Z, Perl Y, Morrey CP, Xu J. Rule-based support system for multiple UMLS semantic type assignments. J Biomed Inform. 2013;46(1):97–110. 23041716 10.1016/j.jbi.2012.09.007 PMC3563942

[87] Gu HH, Perl Y, Elhanan G, Min H, Zhang L, Peng Y. Auditing concept categorizations in the UMLS. Artif Intell Med. 2004;31(1):29–44. 15182845 10.1016/j.artmed.2004.02.002

[88] He Z, Morrey CP, Perl Y, Elhanan G, Chen L, Chen Y, Geller J. Sculpting the UMLS refined semantic network. Online J Public Health Inf. 2014;6(2). 10.5210/ojphi.v6i2.5412 PMC4235323 25422719

[89] Chen L, Morrey CP, Gu H, Halper M, Perl Y. Modeling multi-typed structurally viewed chemicals with the UMLS refined semantic network. J Am Med Inform Assoc. 2009;16(1):116–31. 18952946 10.1197/jamia.M2604 PMC2605602

[90] Morrey CP, Chen L, Halper M, Perl Y. Resolution of redundant semantic type assignments for organic chemicals in the UMLS. Artif Intell Med. 2011;52(3):141–51. 21646001 10.1016/j.artmed.2011.05.003 PMC3134941

[91] Gu HH, Elhanan G, Perl Y, Hripcsak G, Cimino JJ, Xu J, Chen Y, Geller J, Morrey CP. A study of terminology auditors’ performance for UMLS semantic type assignments. J Biomed Inform. 2012;45(6):1042–8. 22687822 10.1016/j.jbi.2012.05.006 PMC3504177

[92] Toutanova K, Klein D, Manning CD, Singer Y. Feature-Rich Part-of-Speech Tagging with a Cyclic Dependency Network. NAACL2003.

[93] Part-of-speech tagging. https://en.wikipedia.org/wiki/Part-of-speech_tagging (accessed Oct 15, 2021). 2021.

[94] Papagiannopoulou E, Tsoumakas G. A review of keyphrase extraction. Wiley Interdisciplinary Reviews: Data Mining and Knowledge Discovery. 2020;10.

[95] Mihalcea R, Tarau P. TextRank: bringing order into text. EMNLP: Association for Computational Linguistics; 2004: 404–11.

